# Necrophagous Insects and Internal Temperature Synergistically Determine Duration of the Decomposition Process for Deer Carcasses When Vertebrate Scavengers are Excluded

**DOI:** 10.1002/ece3.73476

**Published:** 2026-04-20

**Authors:** Ai Hachiya, Akino Inagaki, Maximilian L. Allen, Yosuke Sembongi, Kahoko Tochigi, Shinsuke Koike

**Affiliations:** ^1^ Faculty of Agricultural Science Tokyo University of Agriculture and Technology Fuchu Japan; ^2^ Institute of Global Innovation Research Tokyo University of Agriculture and Technology Fuchu Japan; ^3^ Illinois Natural History Survey University of Illinois Champaign Illinois USA; ^4^ BOULDER co., Ltd. Minami‐Aizu Japan; ^5^ Biodiversity Division National Institute for Environmental Studies Tsukuba Japan

**Keywords:** body internal temperature, carrion consumption, dipteran larvae, maggot mass heat generation, vertebrate scavenger

## Abstract

Necrophagous Diptera larvae are usually the earliest carrion‐visiting necrophages in terrestrial ecosystems. However, despite the significant contribution of dipteran larvae to the natural decomposition process of large mammals in the ecosystem, which are thought to have a significant impact on ecosystem function and stability, there are still many unknowns regarding the function of maggots during actual decomposition of carcasses in the field. This study investigates the role of necrophagous dipteran larvae in the decomposition of sika deer ( *Cervus nippon* ) carcasses in a temperate forest ecosystem while excluding vertebrate scavengers. We evaluated the effects of ambient internal temperatures and body weight on carcass decomposition. A total of 12 deer carcasses were monitored for weight loss and both temperature changes from September 2021 to June 2023. The findings indicate that the average time for a deer carcass to decompose to 30% of its original weight was approximately 14 days, significantly influenced by seasonal temperature variations and initial carcass weight. Internal temperatures within carcasses were observed to exceed ambient temperatures by 10°C–30°C. Furthermore, the shorter the duration after carcass placement for the body's internal temperature to rise significantly above the air temperature, the faster the body decomposes. These results support our hypothesis that metabolic heat generated by maggot masses enhances larval activity and accelerates decomposition. This study also reveals that while Diptera larvae can significantly contribute to decomposition under optimal conditions, vertebrate scavengers play an essential role in the overall decomposition process, particularly in colder months.

## Graphical abstract

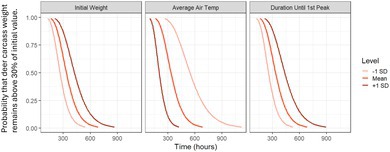

## 1 Introduction

In terrestrial ecosystems, vertebrate carcasses occur spontaneously in time and space, and are typically decomposed quickly through consumption by a variety of organisms, including microorganisms, invertebrates such as Diptera, Coleoptera, and Hymenoptera, and vertebrates, such as vultures and carnivores. Generally, vertebrates have a significant impact on the decomposition of carcasses by visiting carcasses and consuming large amounts of carrion in a short period (e.g., Sebastián‐González et al. 2020, 2021). On the other hand, invertebrates, especially insects, not only inevitably visit carcasses in their natural habitat, but are also usually the earliest carrion‐visiting necrophages in terrestrial ecosystems (Smith 1986; Benbow et al. 2013), contributing significantly to carcass decomposition alongside vertebrates (Anderson et al. 2019).

The invertebrate decomposition of animal carcasses follows a predictable sequence known as insect succession, where different species arrive in “waves” as the biological and chemical state of the carcass changes (e.g., Matuszewski and Mądra‐Bielewicz 2022; McIntyre et al. 2024; Hans and Weidner 2025). In the Fresh Stage, pioneer colonizers—including blow flies (Calliphoridae) and house flies (Muscidae)—are often the first to arrive. These pioneer colonizers frequently arrive within minutes or hours of death and lay eggs in openings of the carcass (i.e., natural orifices or wounds; Galante and Marcos‐Garcia 2008). Later, during the Bloat and Active Decay Stage, active decomposers—including flesh flies (Sarcophagidae)—arrive and often deposit live larvae in and on the carcass. During these stages, massive populations of maggots consume the majority of the soft tissue, sometimes removing up to 90% of carcass biomass in under a week (Galante and Marcos‐Garcia 2008). In the later Advanced and Dry Decay Stage, as the carcass dries, dermestid beetles (hide beetles) and clerid beetles (ham beetles) appear to feed on tougher materials like skin, tendons, and hair. Cheese flies (Piophilidae) may also arrive during the later stages of butyric fermentation (e.g., Pechal et al. 2014; Bansode et al. 2025).

Diptera includes blowflies (Calliphoridae), which are often among the first colonizers, and display high metabolic and physiological sensitivity to ambient temperature changes (Anderson and Gemmellaro 2025). This is most obvious in the larval stages, since the time taken to reach a certain life stage or size depends directly on the temperature to which an individual is exposed (Amendt and Lutz 2025). For example, lower temperatures decrease activity, halt oviposition in adults, and reduce egg and larval survival, as well as reduce larval growth rates (Charabidze 2025; Rivers and O'Reilly 2025). Therefore, the rate at which dipteran larvae decompose carcasses is also highly temperature‐dependent, with warmer temperatures increasing the probability of colonization and accelerating decomposition progression (Waters and Benbow 2025). Therefore, in temperate regions, it is thought that vertebrates are the main scavengers during the cold season when the temperature drops below a certain level (Inagaki et al. 2025), and that both vertebrates and invertebrates function as the main scavengers during the warm season.

In the Diptera larval decomposition of carcasses, the internal temperature of the carcasses and the ambient air temperature also influence the rate of decomposition (e.g., Barton et al. 2021; Waters and Benbow 2025). The most notable contributor to internal temperatures is metabolic thermogenesis by bacterial communities and fly maggot masses (larval aggregations) (Gbenonsi and Higley 2025). Internal temperatures of maggot masses can vary considerably from ambient and ground conditions and, in some cases, have been reported to exceed the surrounding environment by as much as 10°C –30°C (Gbenonsi and Higley 2025; Waters and Benbow 2025). Additionally, repeated temperature increases during the active feeding of maggot masses within the carcass further stimulate maggot activity (Charabidzé and Aubernon 2023). For example, *Lucilia cuprina* larvae are capable of regulating their body temperatures within a carcass to facilitate their development, and increased temperatures among the larvae of these species are apparently facilitated and maintained by the larvae forming large maggot masses, where they aggregate during feeding and development (Amendt and Lutz 2025). It has been speculated that individuals cycle in and out of such masses in order to achieve the optimal temperatures for development (Amendt and Lutz 2025; Gbenonsi and Higley 2025).

There have been numerous previous forensic entomology studies of carcass decomposition by necrophagous dipteran larvae (e.g., Ahmad and Omar 2018; Barton et al. 2021; Wallace 2025; Matuszewski 2026). The physiological function of maggot mass heat generation has often been evaluated, but little has been done from an ecological perspective. Furthermore, while much of this research has been conducted in Europe and North America, there are limited examples in temperate Asia (e.g., Ishihara and Iwase 2020; Urabe et al. 2022). Maggot mass heat generation testing has also mostly been conducted in laboratory experiments where environmental conditions can be kept constant, or using small, easily managed animals' carcasses. However, despite the significant contribution of dipteran larvae to the natural decomposition process of large mammals in the ecosystem, which are thought to have a significant impact on ecosystem function and stability (Inagaki et al. 2025), there are still many unknowns regarding the function of maggots during actual decomposition of carcasses in the field. This includes the relationship between temperature and decomposition, the dynamics of temperature changes in maggot masses within the carcasses, the dynamics of the maggots that generate them, and the process of carcasses consumption. This is because wild mammal carcasses are visited by a variety of vertebrate and invertebrate scavengers, often making it difficult to evaluate the decomposition function of insects alone.

In this study, our aim was to evaluate the function of necrophagous dipteran larvae during the decomposition process of sika deer ( *Cervus nippon* ) carcasses while in the field in temperate Asian forests. The novelty of our study was using an experimental area where large and middle vertebrate scavengers were excluded in the field. We also recorded the consumption time (change in body weight) of wild large mammal carcasses, as well as changes in the internal temperature of the carcasses and air temperature. Furthermore, for some deer carcasses, we recorded the dispersal of maggots contributing to decomposition to examine the relationship between dispersal maggot number, internal temperature of carcasses, and changes in the body weight of the deer carcasses in order to clarify the relationship between maggot dynamics and the influence of internal temperature on the carcass. A previous study has shown that air temperature and internal temperature of carcass affect the activity of maggot decomposition (Rivers et al. 2011). Therefore, we hypothesized that the consumption time of large mammal carcasses caused by maggots alone would be shorter if the carcass weight was lighter, the duration of internal temperature until sudden rise was shorter, and the average air temperature was higher. We discuss our findings with reference to how temperature variation across a carcass might affect decomposition rates and the impact of maggots on seasonal deer carcass decomposition, as well as examine the roles of scavenging in temperate forest ecosystems by clarifying the relationship between the carrion consumption of vertebrates and invertebrates.

## 2 Material and Methods

### 2.1 Study Area

We conducted our research at Minami‐Aizu town, Fukushima Prefecture, in central Japan (N37°04′01.02″, E139°40′22.19″). The mean annual temperature was 10.2°C and the mean annual rainfall was 1116.1 mm in Tajima weather observation points far from 18 km (Japan Meteorological Agency, 2025: http://www.data.jma.go.jp/gmd/risk/obsdl/index.php). The forest types included deciduous broadleaved forests (comprised mainly of *Quercus serrata* , *Cerasus leveilleana*, and *Juglans ailanthifolia* ). The forest floor consisted primarily of bamboo grasses.

There are no obligate mammalian predators of large herbivores in the study area, so the sources of carrion from large mammals available to vertebrate scavengers are mostly natural‐caused deaths (e.g., disease, starvation, and neonatal predation) or human‐caused deaths (e.g., hunting and culling) rather than predation. The main mammalian scavengers at ungulate carcasses known near this study area are Asian black bear ( *Ursus thibetanus* ), wild boar ( *Sus scrofa* ), red fox ( *Vulpes vulpes* ), raccoon dog (*Nyctereutes viverrinus*), masked palm civet ( *Paguma larvata* ), and Japanese marten ( *Martes melampus* ). The main avian scavengers are the jungle crow ( *Corvus macrorhynchos* ), black kite ( *Milvus migrans* ), and mountain hawk‐eagle ( *Nisaetus nipalensis* ) (Inagaki et al. 2020; Koike 2025). But we excluded vertebrate scavengers in this study (see details below).

### 2.2 Target Necrophagous Dipteran

We collected ten larvae from each deer during field visit and transferred them to a plastic container. We reared the collected living samples on vermiculite or sawdust in a disposable semi‐closed plastic container. The larvae were left to develop until the adult stage for identification. We collected the emerged adult flies and identified them using an adult Diptera taxonomic keys (Tanaka 2000; Saigusa 2008; Sasakawa 2013).

We collected mostly *Calliphora vicina* (85%), and also recorded *Lucilia illustris* (10%) and *L. caesar* (5%). Thus, the target study organisms (necrophagous Dipteran larvae) are primarily those of the *Calliphoridae* families. *Calliphoridae*, in particular, are known for being the first to visit a carcass after an animal's death and for contributing significantly to the decomposition of carrion (Smith 1986; Anderson et al. 2019). These Dipteran larvae lay eggs or larvae on the carcass, and the larvae feed on decaying soft tissues for nutrition as they develop. Growth rates vary depending on temperature, but at 20°C indoors, eggs hatch in about one day, and the larvae feed on carrion and reach their final third instar stage in about a week. They then disperse en masse from the carcass to a dry location to pupate (Byrd and Tomberlin 2020).

### 2.3 Permits of Carcass Handling

We obtained sika deer carcasses from culling efforts by snare traps to prevent overabundance and agricultural or ecological damage. Captured deer were euthanized by electrocution quickly after capturing. This method is thought to most minimize pain and distress, and so was used in accordance with the “Welfare and Management of Animals Act” (Ministry of the Environment) and “Specified Wildlife Conservation and Management Plan” (Fukushima Prefecture). We handled deer carcasses according to the guidelines of the American Society of Mammalogists (Sikes and Animal Care and Use Committee of the American Society of Mammalogists, 2016) and the guidelines for animal research set forth by The Mammalogical Society of Japan (2009).

### 2.4 Data Collection

We placed the experimental deer carcasses in the field between May and November, starting in September 2021 and ending in June 2023. We placed deer carcasses at the experimental sites within 6 h of capture to ensure freshness and our ability to document the entire decomposition process. We first recorded the weight of deer carcasses, and then placed them on a 110 × 130 cm wooden board placed on a digital balance (SE‐150KBL‐K; A&D Co. Ltd.). The board was coated with water‐resistant paint to prevent weight changes due to rain. To prevent vertebrate scavengers from scavenging on deer carcasses, we installed an electric fence around the study site, and an iron cage surrounded by metal mesh within the electric fence. The iron cage measured 2 m long × 3 m wide × 2 m in height and consisted of a 10 cm mesh grid. We installed the carcass atop a digital balance inside of the iron cage, and a polycarbonate corrugated sheet was installed on top of the iron cage to minimize bird intrusion from above and the impact of rain on carcass decomposition and carcass weight changes (Figure S1). The experimental sites were a pair of locations set 100 m apart in a straight line in a closed canopy area.

To determine the amount of weight lost by the deer carcasses, we recorded their weight every hour from the time we placed the carcasses until the end of the experiment. We terminated the experiment when the carcass was decomposed, which we standardized by using when the weight of a carcass reached 30% of its initial weight. We used 30% because (a) terrestrial mammalian bones generally account for approximately 15%–20% of body weight (e.g., Berg and Butterfield 1966); (b) deer have a large rumen within their abdominal cavity that weighs approximately 5%–10% of body weight (Nagy and Regelin 1975), which often contains undecomposed plant matter, which is assumed not to be decomposed by scavengers; and (c) because deer have antlers that can be 1%–5% of their body weight (Huxley 1931) (Figure S2).

In addition, to monitor the internal temperature of the carcasses, we placed waterproof thermometers with loggers (Thermochron G type, KM laboratories Co. Ltd.) in the hind legs, front legs, abdominal cavity, and back, because it is known that temperature changes vary from place to place within the carcass (Barton et al. 2021). To install each thermometer, we made an approximately 5 cm incision, inserted the thermometer under the skin, and then closed the wound with a stapler. Temperatures were recorded every hour after placement. The temperature loggers (Ondotori RTR503B, T&D Co. Ltd.) were retrieved at the end of the experiment. We also installed temperature loggers at the experimental site, away from direct sunlight, to record the air temperature every hour.

For three of the deer carcasses (ID2201, ID2202, and ID2203; Table 1), we used maggot traps to collect final‐stage maggot larvae that had gathered around the deer carcasses, taking advantage of the tendency of maggot larvae to disperse from the carcass as they pupate. The traps were plastic containers measuring 10 cm long × 9 cm wide × 15 cm high filled with 5 cm of water. We placed ten traps randomly around the edge of the wooden board on which the deer carcass was placed, and maggots that fell from the board were collected. Because the trap width covered a total of 100 cm of the circumference of the wooden board (480 cm), we estimated the total number of maggots by multiplying the number of larvae finally collected by 4.8. We collected the maggots in the traps three times a day during the dispersal period at 6:00, 12:00, and 18:00, and if the number of dispersing individuals was significantly high, we collected them as needed. We stored the collected individuals in 80% ethanol and later counted them.

**TABLE 1:** A list of the deer carcasses set, including their weight at the time of set, the date of placement, the average outside temperature during the placement period (at hourly intervals), the time it took for the temperature measured on the thermometer placed inside the deer to significantly exceed the outside temperature, the temperature at which the internal temperature reached its highest recorded temperature and the outside temperature at that time, and the time it took for the deer to completely decompose (i.e., reach 30% of its original weight).

Carcasses ID	Body weight (kg)	Set day	Average temperature	Duration of 1st peak inside temperature (h)	The highest internal temperature (air temperature at that time)		Duration of defecate carcass (h)
2101	21.9	2021/9/7	16.5	127	34.3	(12.6)	235
2102	19.8	2021/9/22	15.2	102	39.5	(17.6)	178
2103	21.5	2021/10/12	7.7	122	28	(13.1)	802
2104	29.3	2021/11/9	4.2	n/a	n/a		n/a
2201	60.7	2022/5/26	16.1	172	32	(28.5)	862
2202	35.9	2022/6/14	18.1	279	42.5	(16.1)	357
2203	18.5	2022/7/25	21.5	85	28.5	(27.2)	149
2204	23.0	2022/9/5	18.7	69	44.5	(23.4)	167
2205	46.9	2022/9/18	16.1	104	40.5	(20.2)	194
2301	65.0	2023/6/8	16.5	152	44	(23.9)	427
2302	39.6	2023/6/9	17.1	174	38.5	(25.8)	481
2303	54.8	2023/6/28	19	74	40.8	(18.3)	214

### 2.5 Statistical Analyses

To examine how the time until carcass mass declined varied with initial carcass mass, air temperature, and internal carcass temperature, we used an accelerated failure time (AFT) model. This is a parametric survival analysis framework that evaluates how covariates influence the timing of events through survival regression models (Wei 1992). In the AFT model, the event time (hours) was defined as the first observation at which carcass mass fell below 30% of its initial value. Carcasses that did not reach this threshold during the monitoring period were treated as right‐censored at their last recorded observation (only ID2104). Covariates included initial body weight (kg), mean air temperature during the monitoring period (°C), and the duration until the first peak in internal temperature (hours). We standardized all covariates prior to analysis to allow comparisons of effect sizes. We fitted the models using a log‐normal distribution, which provided a substantially better fit (lowest Akaike Information Criterion; Burnham and Anderson 2002) than other commonly used AFT distributions (Weibull, Gaussian, logistic, and log‐logistic). We created AFT models and conducted all analyses using R version 4.5.1 (R Core Team 2025) with the survival package (Therneau 2024). We generated predicted survival curves at low (−1 SD), mean, and high (+1 SD) values of each covariate. For initial body weight, these values corresponded to approximately 19.6 kg, 36.4 kg, and 53.3 kg. For average air temperature, the values were 10.7°C (low), 15.6°C (mean), and 20.4°C (high). For the duration until the first peak in internal carcass temperature, the values were 72.1, 133.0, and 193.0 days, respectively.

In this study, we calculated internal body temperature as the average of the thermometers installed with usable data. Furthermore, since the collected data suggested that the body temperature during life would have an effect for the first 24 h after installation, we calculated the average temperature for the duration of the survey from the average temperature starting 25 h after installation. Furthermore, when we compared the internal body temperature with the air temperature at that time, the average internal body temperature relative to the air temperature was 1.15 ± 0.24 SD times higher. Therefore, if the internal body temperature was 1.39 times the air temperature at that time, we assumed that the internal body temperature was changing at a rate greater than normal. Therefore, we defined the first internal body temperature peak as the time when the internal body temperature exceeded 1.4 times the air temperature for the first time after the carcass was placed.

## 3 Results

We placed a total of 12 deer carcasses, and maggots were collected from three of them (ID2201, ID2202, and ID2203), placed in May, June, and July (Table 1). Of the 12 carcasses, we observed the carcass placed in November for 30 days after placement, but discontinued observation then because no decomposition was observed and snowfall began (Figure S3). The following analyses excluded this carcass and focused on the 11 other carcasses (Table 1).

Internal body temperatures were consistently recorded higher in the abdominal cavity and back than in the hind legs and front legs. Maggot masses were also observed more frequently in the torso.

The first peak in internal temperature occurred on average 132.7 ± 57.8 h after placement, with the shortest duration being 69 h placed in September (ID2204) and the longest duration being 279 h placed in June (ID2202). The highest average internal temperature was 37.5°C ± 5.7°C, with the highest individual temperature being 44.5°C for the carcass placed in September (ID2204).

**FIGURE 1:**
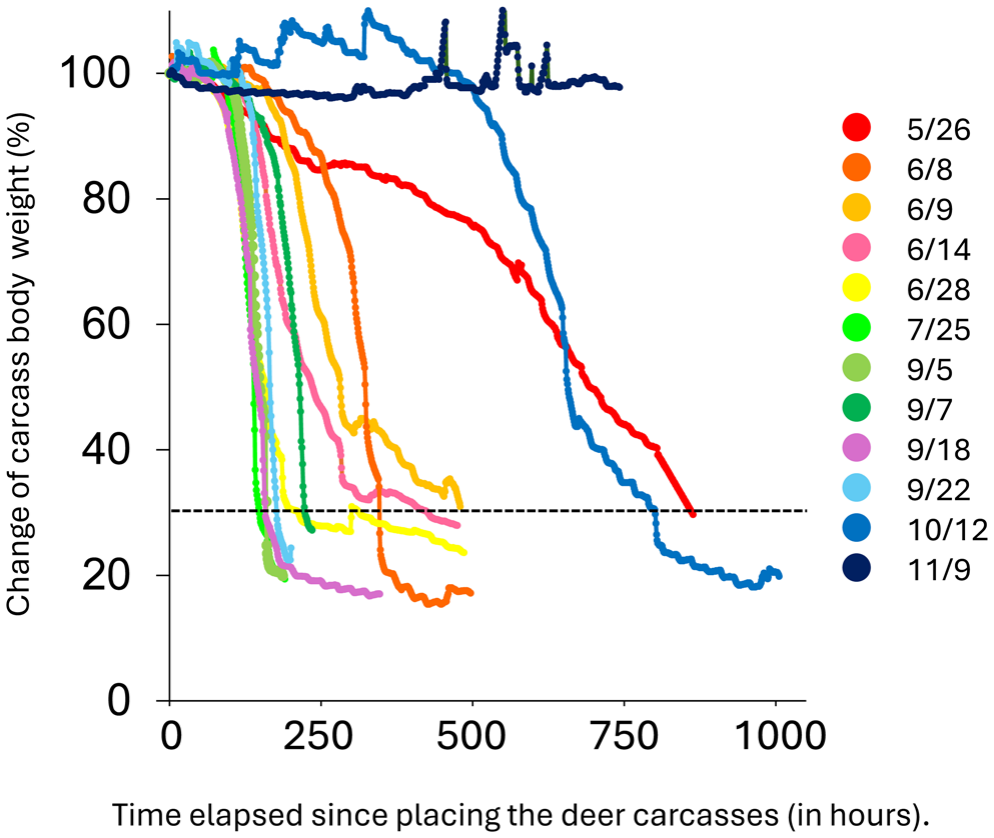
Weight loss rate of deer carcasses and time (hour) elapsed since placement. The date in the legend indicates the date the deer carcasses were set.

The duration it took for the deer's weight to reach 30% of its original weight was 369.6 ± 242.1 h, with the shortest time being 149 h when the carcass was placed in July (ID2203), and the longest being 862 h when the carcass was placed in May (ID2201) (Figure 1). While carcasses placed in October and May took more than 30 days to be consumed, carcasses placed between June and September were consumed after an average of 11 days.

**FIGURE 2:**
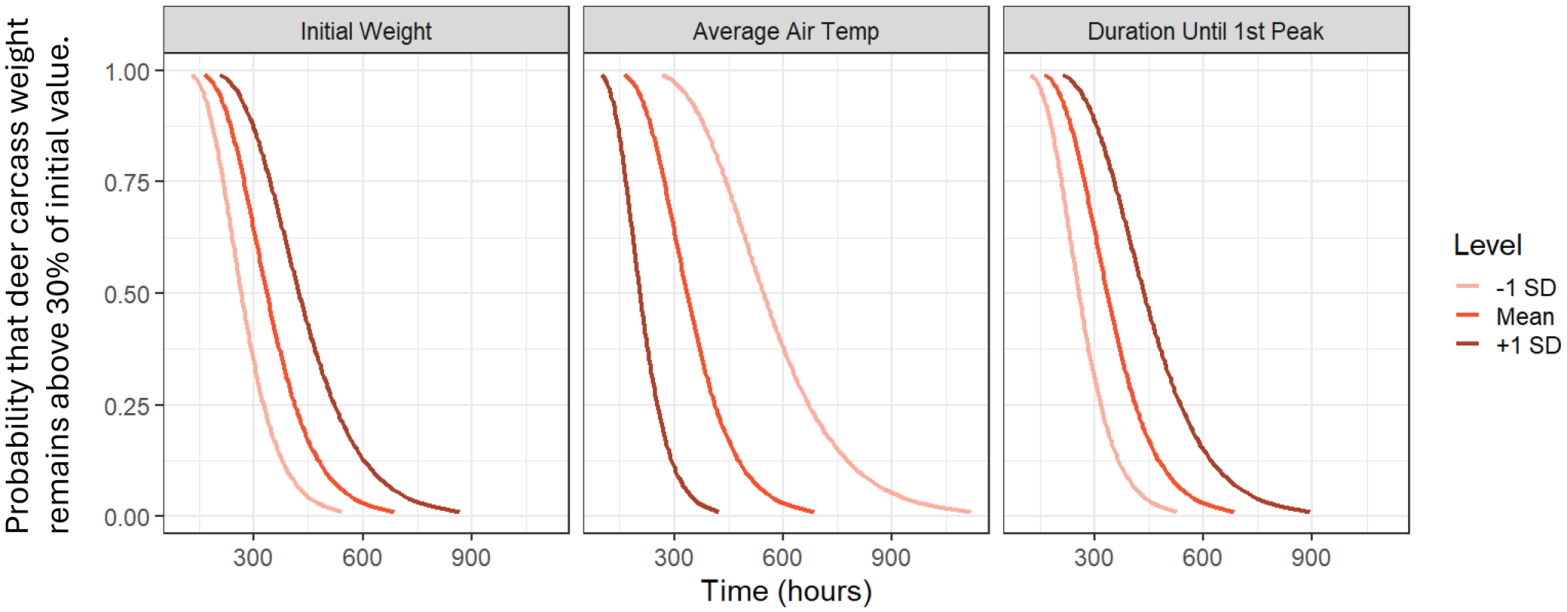
Predicted survival curves from the accelerated failure time (AFT) model showing the probability that deer carcass weight remains above 30% of its initial value over time. Curves represent predictions at −1 SD, mean, and + 1 SD of each covariate (initial body weight, average air temperature, and duration until the first peak in internal carcass temperature), while holding other covariates at their mean values.

The AFT model showed that all three covariates significantly affected the consumption time of carcasses (*p* < 0.05; Table 2). Greater initial body mass increased consumption time by 26.5% (*p* = 0.016), and a longer time until the first peak in internal carcass temperature increased consumption time by 30.2% (*p* < 0.001). In contrast, higher average air temperature decreased consumption time by 38.6% (*p* = 0.009) (Table 2). Essentially, carcasses were consumed more quickly when they were lighter, exposed to higher air temperatures, or reached the first peak in internal temperature sooner (Figure 2; Table 2).

**TABLE 2:** Result from the accelerated failure time (AFT) model investigating the covariates affecting consumption time of deer carcass. A positive *β* implies an increase in consumption time with exp (*β*) > 1. A negative *β* indicates a reduction in consumption time, with exp (*β*) < 1. SE denotes the standard error of the model coefficients.

Covariates	Coefficient	exp (*β*)	SE	*z* value	*P* value
Initial body weight	0.235	1.265	0.098	2.410	0.016
Average temperature	−0.487	0.614	0.139	−3.510	< 0.001
Duration until first peak in internal temperature	0.264	1.302	0.102	2.600	0.009

**FIGURE 3:**
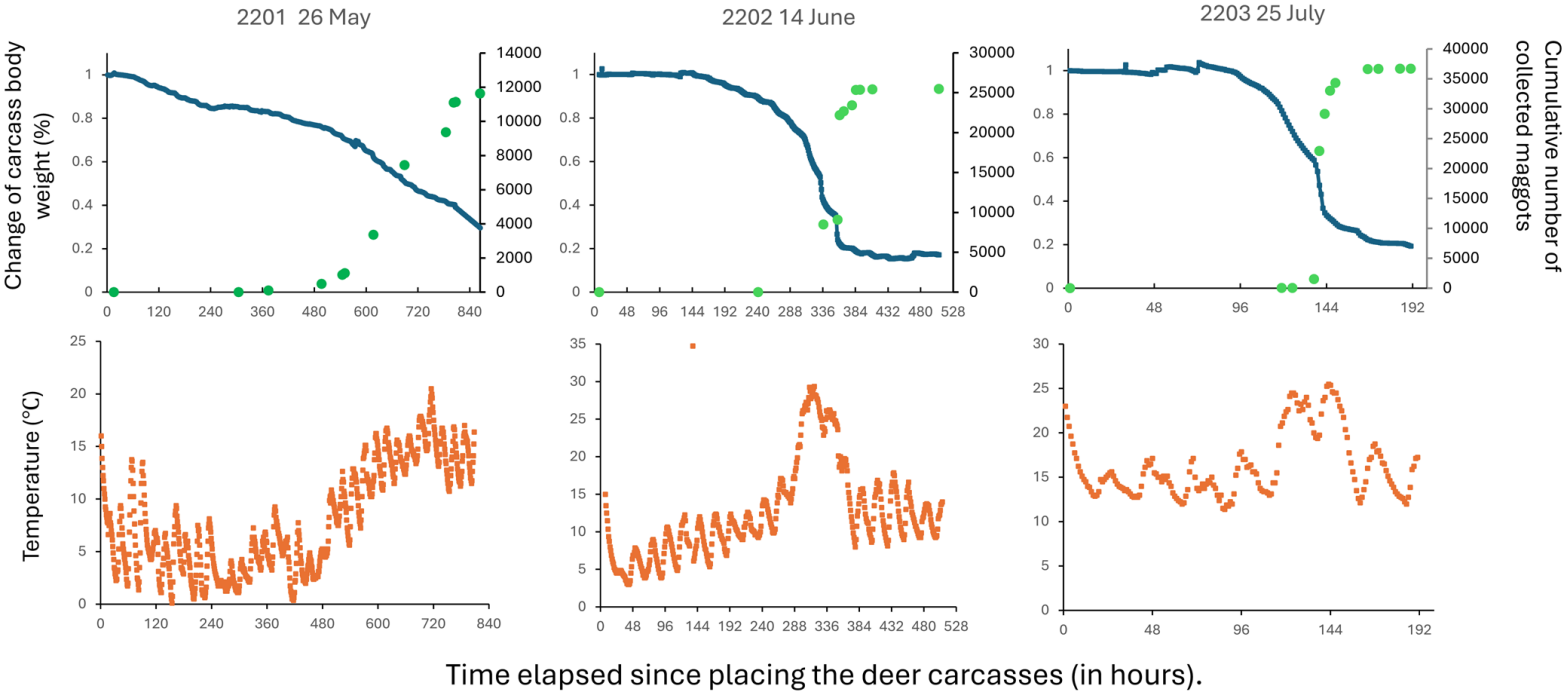
The relationship between the body weight of deer carcasses from which dispersed maggots collected (ID: 2201, 2202, and 2203 and set day), (blue), the cumulative number of maggots collected (green), the internal temperature of the deer carcasses (orange), and the time (hour) elapsed since the deer carcasses were set.

For carcasses from which we collected maggots, we first collected maggots 480 h after placement from deer placed in May (2201), 305 h from deer placed in June (ID2202), and 125 h from deer placed in July (ID2203). We collected a total of 11,631 maggots from the deer placed in May, 25,465 from deer placed in June, and 36,672 from deer placed in July, giving an estimated total of 558,288, 122,232, and 176,036 maggots, respectively. When maggot dispersal began, the deer carcass also experienced a significant loss of body weight, and a sudden drop in body temperature was observed within the carcasses (Figure 3).

## 4 Discussion

Dipteran larvae play an important role as carrion consumers, consuming most of the soft tissue of carcasses when necrophagous vertebrates are absent (e.g., Anderson et al. 2019; Byrd and Tomberlin 2020). However, there has been no quantitative evaluation of the extent to which they contribute to the decomposition of large vertebrate carcasses in the wild, which has a significant impact on nutrient cycling in ecosystems. This study has shown that although the decomposition process of deer carcasses by dipteran larvae is strongly affected by seasonal temperature differences, in temperate forests, dipteran larvae alone can decompose a deer carcass in about 10 days under certain temperature conditions.

In our study, we also observed that the internal body temperature exceeded the ambient temperature by 10°C–30°C, confirming that heat was generated by maggot masses in the field, as in previous laboratory experiments (e.g., Johnson et al. 2014; Heaton et al. 2014). Furthermore, it was suggested that the body temperature may also increase due to the presence of maggot masses. A key finding of our study was that the shorter the duration it took for internal temperatures to reach their peak, the shorter the duration it took for the deer carcass to be consumed. The heat generated by the maggot mass is believed to be beneficial for larvae because it maximizes their feeding efficiency by producing sufficient proteolytic enzymes for tissue breakdown (Richards et al. 2009; Ireland and Turner 2006; Goodbrod and Goff 1990; Schoofs et al. 2009). By maintaining a stable microclimate with increased temperatures, larvae are also protected against any chilling or cold shock injury brought on by sudden and temporary drops in ambient temperature—and this has been supported by Huntington et al. (2007). In any case, it can be said that repeated increases in temperature accelerate the decomposition of maggots. Consequently, it can be said that rising internal temperatures accelerate the generation of metabolic heat in maggots, thereby accelerating the consumption time of the deer carcass, supporting our initial hypothesis.

The relationship between the heat generated by the maggot mass and the maggot dynamics was revealed by comparing the decomposition process of the three individuals from which we collected dispersal larvae and the temperature changes in the carcasses. With the exception of the May trial, for which no clear internal temperature rise was observed, the maggots' internal temperatures rose several times, and the deer's weight decreased significantly at almost the same time as they reached their maximum temperature and the maggots began to disperse. This result suggests that repeated internal temperature rises increased the average internal temperature, which in turn increased the overall feeding rate and larval growth rate, resulting in a rapid progression of the larval developmental stages and promoting dispersal. In any case, whether dispersal occurs all at once or over a long, continuous period, it can have a significant impact on the rate at which a carcass decomposes.

The average time required for carcasses to be consumed by invertebrates was 14 days, or 11 days when excluding carcasses placed in May and October which took longer to decompose. Carcasses placed between late June and September took less than 10 days to be consumed. These differences appear to be closely related to carcass weight and temperature. First, a previous study using 45 pigs ( *Sus scrofa* ) weighing 3.2–82 kg reported that the heavier the carcass, the longer the decomposition time; our study also demonstrated a longer decomposition duration with increasing carcass weight, consistent with a previous study (Sutherland et al. 2013). Second, because rising air temperatures likely contributed to increased maggot activity associated with the maggot masses, carcasses placed in May and October, when temperatures were low, likely took longer to be consumed. Conversely, high temperatures accelerated decomposition, particularly during the summer. However, in some cases, such as the June cases (ID2301 and ID2302) where the carcasses were placed at roughly the same time, decomposition was faster despite the heavier body weight of 2301. This suggests that minor meteorological factors, such as rainfall and temporary drops in temperature, may also play a role, which cannot be explained by temperature alone.

In this study, we measured the decomposition process of deer carcasses using only maggots while excluding vertebrate scavengers. A previous study investigating the decomposition process of 44 deer carcasses placed near the study site between June and November, including vertebrates, found that deer carcasses were consumed in an average of seven days (Inagaki et al. 2022). Since the carcasses placed between May and October in this study took approximately 14 days to be consumed, the presence of vertebrate scavengers can be inferred to have accelerated the consumption rate by approximately seven days. This finding further demonstrates that the presence of vertebrate scavengers is likely essential for carcass consumption from October onwards through early June, when maggot function slows down dramatically. Despite the absence of specialized carrion scavengers like vultures, Japan's forests have a sufficiently high carrion removal capacity, suggesting that healthy ecosystem services are being provided. The results of this study demonstrate that flies, which inevitably visit carrion, alone cannot fully fulfill this function, reaffirming the importance of vertebrate scavengers in ecosystems.

However, this study has several limitations. First, it cannot verify potential forensic or entomotoxicological aspects. Firstly, the deer used in this experiment were electrocuted, which differs from the actual causes of death in the wild. Different manners of death may influence decomposition dynamics and insect colonization patterns. For example, previous studies have shown that the manner of death can affect the diversity, succession, and colonization of forensic entomofauna (Farag et al. 2021). Therefore, there is a possibility that the results of this study do not accurately reflect the actual decomposition process in the wild. The second point is that, although only blowflies were collected in this study, the functions of other carrion‐eating insects could not be evaluated. Since *Fannia prisca* has been identified as a decomposer in Japan (Urabe et al. 2022), it will be necessary to investigate the potential roles of other carrion‐eating insects, such as Diptera and Coleoptera, in the future. Furthermore, this point could influence potential interspecies interactions. For example, the potential role of predators and competitors of larvae, which may significantly influence decomposition dynamics and insect succession. Predatory or competitive insects, particularly Coleoptera, can interact with Diptera larvae and modify the structure of necrophagous communities. For example, silphid beetles such as Necrodes spp. are known to compete with blowflies for access to large vertebrate carrions and can influence larval abundance and development (Matuszewski and Mądra‐Bielewicz 2022). Also, while medium and large vertebrates were excluded in this study, small mammals or birds may still access the carcass through the mesh and potentially affect larval populations. In the future, considering these ecological interactions would provide a more comprehensive interpretation of the decomposition process and the observed insect assemblies.

This study quantitatively demonstrated that carrion feeding by invertebrates also significantly contributes to carrion consumption in ecosystems during warm seasons. Future research will be needed to clarify the relationship between carrion consumption by vertebrate scavengers and invertebrate scavengers, such as the amount of deer carcasses consumed by each species. Research into the types of scavengers that utilize animal carcasses and the ecological roles they play has been limited to specific regional cases, leaving many unresolved issues regarding the complex relationships between species or communities that arise from animal carcasses. It is hoped that such basic research in a variety of ecosystems (environments) and animal species will lead to a better understanding of the role of vertebrate and invertebrate scavengers in maintaining healthy ecosystems.

## Author Contributions

**Ai Hachiya:** data curation (equal), investigation (equal), methodology (equal), writing – original draft (equal). **Akino Inagaki:** conceptualization (equal), data curation (equal), formal analysis (equal), funding acquisition (equal), investigation (equal), methodology (equal), project administration (equal), supervision (equal), visualization (equal), writing – original draft (equal). **Maximilian L. Allen:** writing – review and editing (equal). **Yosuke Sembongi:** resources (equal). **Kahoko Tochigi:** formal analysis (equal), visualization (equal). **Shinsuke Koike:** conceptualization (equal), data curation (equal), formal analysis (equal), funding acquisition (equal), investigation (equal), methodology (equal), project administration (equal), supervision (equal), visualization (equal), writing – original draft (equal).

## Funding

This work was partly supported by JSPS KAKENHI Grant Numbers, 21K19858, 21J20185, and 24K23917 and Institute of Global Innovation Research, Tokyo University of Agriculture and Technology.

## Conflicts of Interest

The authors declare no conflicts of interest.

## Supporting information


**Figure S1:** A distant view of the study site. An iron cage is set up within an electric fence. A digital scale is placed inside the cage, and a waterproof wooden board is placed on top of that, and a deer carcass is placed on top of that.

**Figure S2:** The decomposition process of a deer carcass. The decomposition process of the carcasses was visually decided as follows: Flesh and bloat stage, in which no maggots were visible on the body surface; Active decay stage, in which maggots were visible on the body surface; Advanced decay stage, in which maggots are active and the body is rapidly decaying; and After advanced decay, in which maggots finish dispersing and ossification is confirmed.

**Figure S3:** The relationship between the body weight of deer carcasses (ID and set day) (blue), the internal temperature of the deer carcasses (orange), air temperature (green), and the time (hour) elapsed since the deer carcasses were set.

## Data Availability

The data analyzed in the manuscript is available as Supporting Information.
