# Supplementary figures and images for: Necrophagous Insects and Internal Temperature Synergistically Determine Duration of the Decomposition Process for Deer Carcasses When Vertebrate Scavengers are Excluded
Source: Ecol Evol. 2026 Apr 20;16(4):e73476. doi: 10.1002/ece3.73476 (PMC13093901; doi:10.1002/ece3.73476)

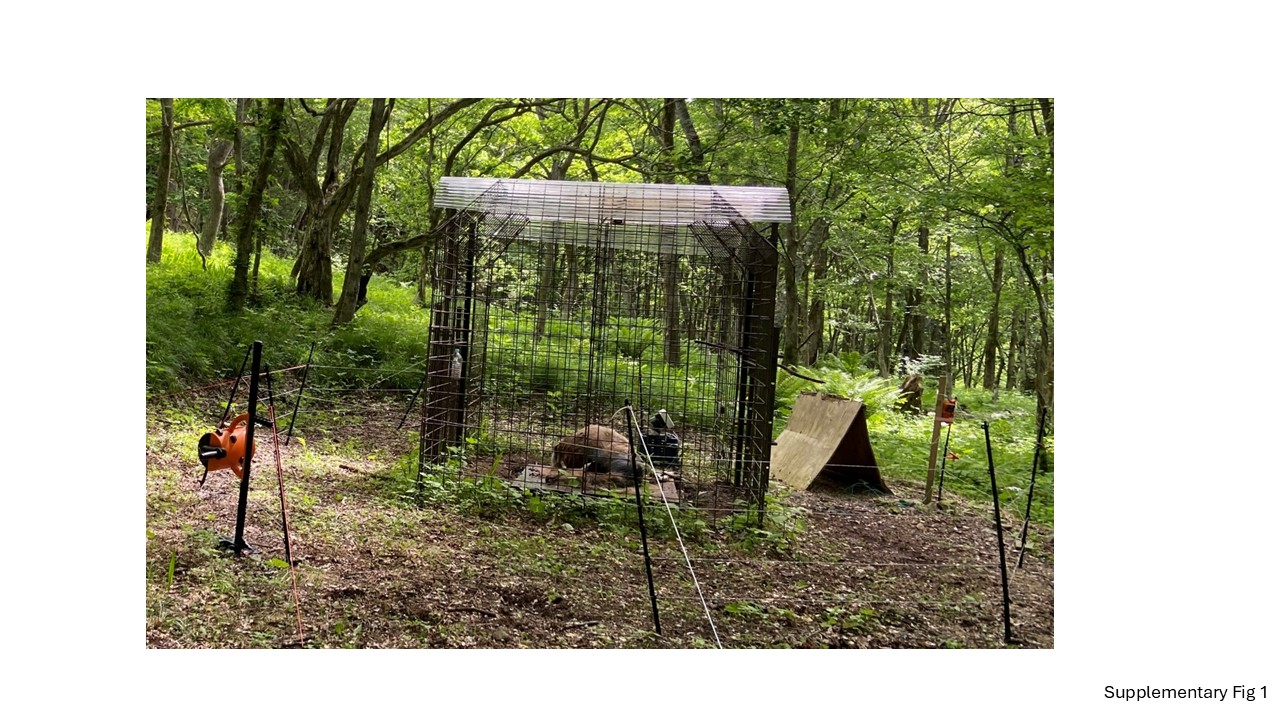

Supplement: Supplementary file 1 — Figure S1: A distant view of the study site. An iron cage is set up within an electric fence. A digital scale is placed inside the cage, and a waterproof wooden board is placed on top of that, and a deer carcass is placed on top of that. [file ECE3-16-e73476-s002.jpg]

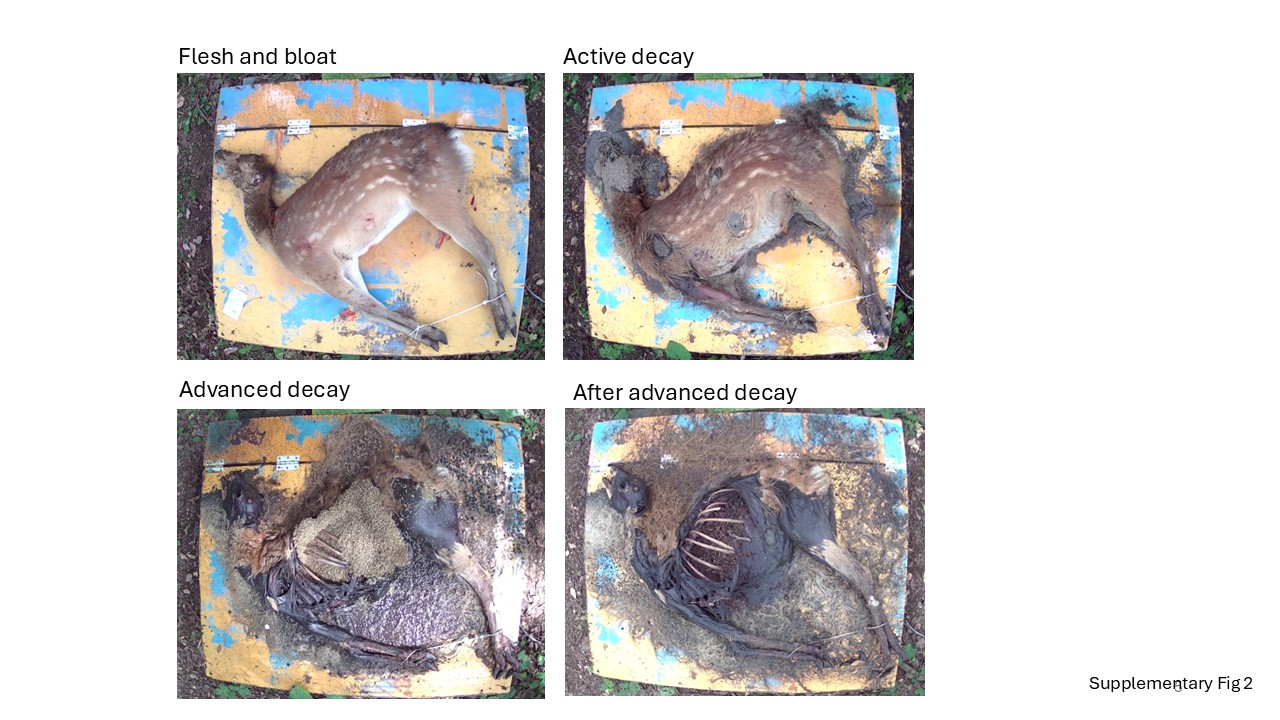

Supplement: Supplementary file 2 — Figure S2: The decomposition process of a deer carcass. The decomposition process of the carcasses was visually decided as follows: Flesh and bloat stage, in which no maggots were visible on the body surface; Active decay stage, in which maggots were visible on the body surface; Advanced decay stage, in which maggots are active and the body is rapidly decaying; and After advanced decay, in which maggots finish dispersing and ossification is confirmed. [file ECE3-16-e73476-s003.jpg]

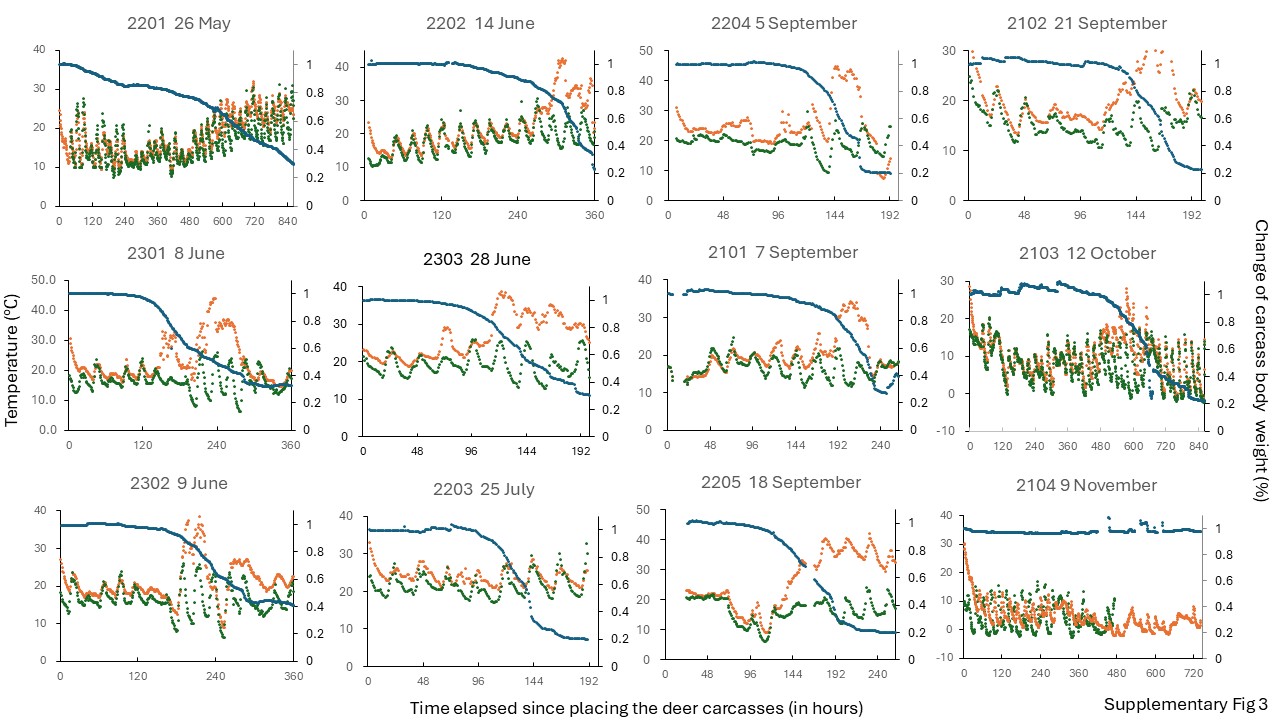

Supplement: Supplementary file 3 — Figure S3: The relationship between the body weight of deer carcasses (ID and set day) (blue), the internal temperature of the deer carcasses (orange), air temperature (green), and the time (hour) elapsed since the deer carcasses were set. [file ECE3-16-e73476-s001.jpg]
